# A surprising new hiding place for a dangerous pathogen

**DOI:** 10.7554/eLife.109706

**Published:** 2025-12-08

**Authors:** Sheetal Gandotra, Yogendra Singh

**Affiliations:** 1 CSIR-Institute of Genomics and Integrative Biology New Delhi India; 2 Institution of Eminence, University of Delhi New Delhi India

**Keywords:** tuberculosis, *Mycobacterium tuberculosis*, metabolism, hepatocytes, Human, Mouse

## Abstract

The bacterium that causes TB can hide in liver cells called hepatocytes, and reprogram their metabolism for its own benefit.

**Related research article** Sarkar B, Singh J, Yadav M, Sharma P, Sharma RD, Singh S, Chandramouli A, Mehdiratta K, Kumar A, Kamat SS, Ghorpade DS, Mohanty D, Kumar D, Gokhale RS. 2025. PPARγ mediated enhanced lipid biogenesis fuels *Mycobacterium tuberculosis* growth in a drug-tolerant hepatocyte environment. eLife **14**:RP103817. doi: 10.7554/eLife.103817.

For more than a century, tuberculosis (TB) has been known as a lung disease that is caused by a species of pathogenic bacteria called *Mycobacterium tuberculosis*. Most infections show no symptoms, but a small fraction of infections become active and, if untreated, can be fatal. Indeed, TB is responsible for over one million deaths every year (Bagcchi, 2023).

TB is spread by people inhaling tiny airborne droplets that carry *M. tuberculosis*, and once inside the lungs, these bacteria settle into macrophages and other immune cells, where they can remain dormant for years – surviving inside the very cells that are meant to kill them. However, *M. tuberculosis* can also slip away from the lungs, travel through the bloodstream, and take up residence in other parts of the body. Clinically, this presents as TB infections in the bones, lymph nodes, or even the brain, though these “extrapulmonary” cases are notoriously hard to diagnose.

Now, in eLife, Rajesh Gokhale and colleagues – including Binayak Sarkar as first author – report the results of a study that reveals an unexpected twist: the liver – the body’s detox powerhouse – may be one of the most welcoming hiding spots for the pathogen that causes TB (Sarkar et al., 2025). And the reason the liver is so welcoming is that in addition to infecting macrophages in the liver, *M. tuberculosis* can also infect cells called hepatocytes, which account for about 80% of the mass of the liver (Figure 1).

**Figure 1.**
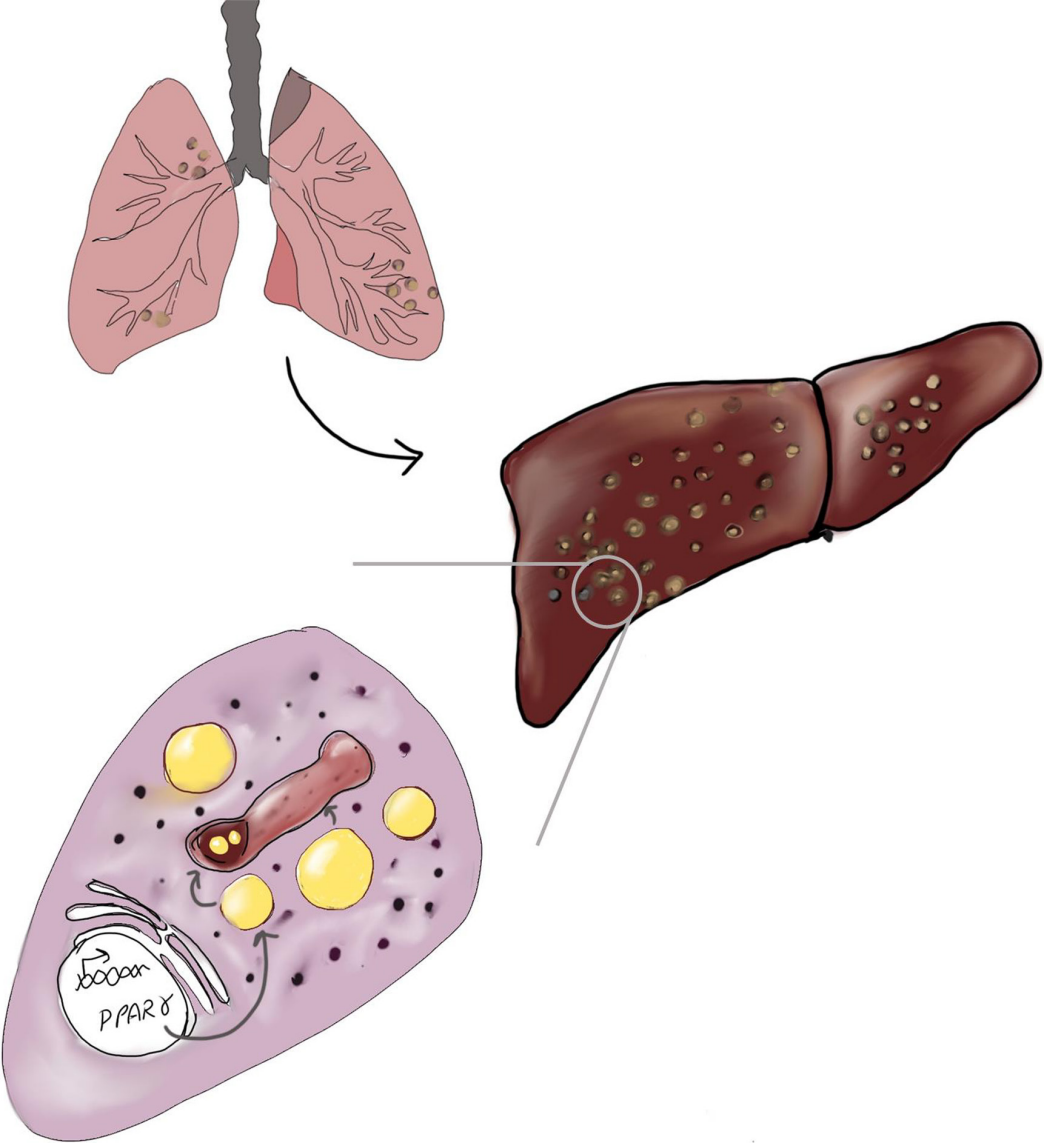
How *M*. *tuberculosis* takes advantage of the liver. *M. tuberculosis*, the pathogen that causes tuberculosis, can travel from the lungs (top left) to the liver (right), where it infects cells called hepatocytes (bottom left) and activates a protein called PPARγ. This encourages the production of lipid droplets (yellow), and the fat inside these droplets helps the pathogen (brown) to survive and even grow inside the liver. The small black structures are secretory vesicles and subcellular organelles.

Sarkar et al. – who are based at the National Institute of Immunology and other institutes in India – report that when *M. tuberculosis* infects hepatocytes, it activates a protein called PPARγ that acts as a master switch for fat production. When PPARγ is activated, it instructs hepatocytes to build lipid droplets that can store fat. To the bacteria, these droplets are like stocked refrigerators, and the fat inside them helps *M. tuberculosis* to survive and even grow inside the liver. Activated PPARγ in macrophages is also involved in helping to defend the body against TB but not through the same pathways as in liver cells (Arnett et al., 2018; Rajaram et al., 2010). Additionally, the lipid droplets in macrophages do not impact bacterial survival like they do in hepatocytes (Jaisinghani et al., 2018; Knight et al., 2018). So liver cells are indeed a different hiding place for *M. tuberculosis*.

The liver is one of the most metabolically active organs in the body. It manages sugars, fats, cholesterol, and amino acids. It also processes nearly every drug we take – including the long, complex regimens used to treat TB. Many TB patients develop liver toxicity during treatment because these drugs put a heavy metabolic burden on the organ. This creates a difficult problem: the very place where drugs are detoxified may also be one of the places where TB hides. The work of Sarkar et al. shows that the infection pushes hepatocytes to increase their production of enzymes that break down drugs. Therefore, while the liver is doing its job of detoxifying medication, the bacteria may be using this metabolic shift to shield themselves from anti-TB drugs.

The new findings suggest that the interaction of *M. tuberculosis* with the liver is not accidental but strategic. The liver’s role in metabolism gives *M. tuberculosis* exactly what it needs: nutrients and the potential to blunt the effects of drug therapy. However, the animal models used by Sarkar et al. are known for exhibiting bacterial dissemination from the lung, where infection can be controlled, to the liver. This means that the latest results are mostly relevant to heavily disseminated tuberculosis, a rarer form of the disease that is often found in immunocompromised individuals. It is also likely that a number of other factors influence how the liver responds to infection by *M. tuberculosis*.

For decades, TB treatment has focused almost entirely on killing the bacterium directly. However, as drug resistance grows – with some patients enduring 18 months of toxic, often painful, drug regimens – scientists are searching for new strategies. The discovery that *M. tuberculosis* can manipulate the metabolism of hepatocytes points to a promising approach: host-directed therapy (Shapira et al., 2024). Instead of only targeting the bacteria, we could block the pathways they exploit, such as those involved in PPARγ activation and lipid droplet formation. By cutting off the pathogen’s food supply, we may weaken it and make existing drugs far more effective. This strategy is already being explored, especially in macrophages (Dawa et al., 2021), and extending it to hepatocytes opens up a new frontier in TB research.

If TB uses fat stores in the liver, could it do the same in other lipid-rich organs? One intriguing possibility is the brain, which is exceptionally rich in lipids and is the site for some of the most dangerous forms of TB. Understanding the relationship between TB and metabolism could reshape how researchers think about persistent infections, drug resistance, and the long-term failure of treatments in some patients. One thing is increasingly clear: TB is not just a lung disease. And solving it may require looking beyond the lungs.
